# The impact of femoral flexion angle and tibial slope on knee gap in total knee arthroplasty

**DOI:** 10.1186/s42836-025-00321-2

**Published:** 2025-07-07

**Authors:** Varah Yuenyongviwat, Chirathit Anusitviwat, Tawan Intiyanaravut, Payap Payapanon, Nimit Thongpulsawasdi

**Affiliations:** 1 Department of Orthopedics, Faculty of Medicine, Prince of Songkla University, Hat Yai, Songkhla, 90110 Thailand; 2 Golden Jubilee Medical Center, Faculty of Medicine Siriraj Hospital, Mahidol University, Nakhon Pathom, 73170 Thailand

**Keywords:** Total knee arthroplasty, Sagittal alignment, Flexion and extension gaps, Femoral flexion angle, Tibial posterior slope

## Abstract

**Background:**

Inadequate gap balance during total knee arthroplasty (TKA) can result in postoperative pain, restricted range of motion, and suboptimal long-term outcomes. The sagittal alignment of the femoral and tibial components plays a pivotal role in determining both the flexion and extension gaps. This study systematically investigates how variations in femoral and tibial sagittal alignment affect knee gap dynamics during TKA, utilizing intraoperative data from a robotic-assisted surgical system.

**Method:**

This retrospective study analyzed data from 40 robotic-assisted TKA procedures. Surgical planning data were obtained using the landmark registration process. The tibial posterior slope was fixed at 3°, while the femoral flexion angle was adjusted incrementally from 3° to 8° in 1° intervals. Medial and lateral flexion gaps were measured at each increment. To examine the effect of tibial posterior slope on knee gap dynamics, the femoral flexion angle was maintained at 3°, and the tibial posterior slope was varied from 3° to 7° in 1° increments. Medial and lateral extension and flexion gaps were recorded for each configuration.

**Results:**

Both medial and lateral flexion gaps progressively increased as the femoral flexion angle was adjusted from 3° to 8°. Similarly, both flexion and extension gaps demonstrated a corresponding increase as the tibial posterior slope was elevated from 3° to 7°. Spearman correlation analysis showed that increasing femoral flexion and tibial slope significantly increased medial and lateral gaps (ρ > 0.99).

**Conclusion:**

Increasing femoral flexion results in a larger flexion gap, while a higher tibial slope leads to proportional increases in both flexion and extension gaps. Future studies incorporating intraoperative validation will be crucial for refining surgical techniques and improving outcomes in TKA.

## Background

Total knee arthroplasty (TKA) is a widely performed surgical procedure designed to alleviate pain and restore function in patients with advanced knee osteoarthritis. [1] The success of TKA relies on accurate prosthetic alignment and careful soft tissue balancing, both of which are critical for optimal biomechanical performance [2]. Inadequate gap balance can compromise patient outcomes by exacerbating postoperative pain, limiting range of motion, and negatively affecting long-term results. [3, 4] Moreover, malalignment of the femoral or tibial components may result in uneven gap dynamics, ultimately leading to joint instability, altered kinematics, accelerated implant wear, and an elevated risk of component loosening and revision surgery [5].

Accurate sagittal alignment is crucial for maintaining knee gap balance and optimizing prosthesis performance. [6] Numerous studies have examined the impact of sagittal component positioning on knee biomechanics and clinical outcomes, with particular focus on posterior tibial slope (PTS) and femoral prosthesis flexion angle. [7] Variations in sagittal alignment have been shown to significantly influence postoperative function, range of motion, and implant longevity. [8–11] Additionally, research highlights that the sagittal positioning of the femoral and tibial components plays a key role in determining flexion and extension gaps, which are essential for postoperative joint stability and overall function [12, 13]

Several studies have examined the influence of sagittal alignment in component positioning on flexion and extension gap dynamics, though the evaluation methods vary across studies. One study assessed the effect of posterior slope adjustments by placing plates on the cut surface of the proximal tibia and using a distraction device to measure the intraoperative gap [14]. Another investigation utilized formalin-fixed cadaveric knees, where CT scans taken in full extension and at 90° flexion were used to create a standardized coordinate system. Virtual bone cuts were then performed with incremental increases in the posterior slope to analyze its effect on gap dynamics [15]. While these approaches have provided valuable insights, recent advancements in robotic-assisted surgery, which offer high accuracy and real-time feedback, now enable more precise intraoperative measurements, potentially yielding more reliable and reproducible data.

Building on this progress, the present study systematically investigates the impact of femoral and tibial sagittal alignment variations on knee gap dynamics during TKA. By utilizing real intraoperative data from the computer planning interface of a robotic-assisted system, this study reduces inter-surgeon variability and enhances measurement accuracy. Gaining deeper insights into the role of sagittal alignment in gap balance will aid in refining alignment strategies, optimizing knee function, and ultimately improving patient outcomes.

## Methods

This retrospective study analyzed data from 40 robotic-assisted TKA procedures performed at our institutions. Surgical data were collected for procedures conducted between September 1st, 2023, and November 30th, 2023. Ethical approval was granted by the local ethics committee and the Institutional Review Board. Patients who underwent robotic-assisted TKA were included. Cases with incomplete medical records or extra-articular deformities were excluded. All patient data were de-identified using coded identifiers. The study primarily focused on knee alignment angles in the sagittal plane and gap measurements in both extension and flexion, recorded in millimeters. Operation planning data were retrieved following the Landmark registration process.

All procedures were performed using the ROSA® robotic-assisted system (Zimmer Biomet, Warsaw, IN, USA) following a standardized surgical protocol. A medial parapatellar approach was employed while preserving the deep medial collateral ligament (MCL). The surgical workflow included anterior tibial exposure, osteophyte excision, and bone registration to evaluate alignment and soft tissue balance. Default surgical parameters consisted of a tibial resection of 10 mm from the lateral tibial plateau perpendicular to the mechanical axis, a medial distal femoral cut of 9 mm perpendicular to the mechanical axis, a posterior condylar axis set to 3° of external rotation, a femoral flexion angle of 3°, and a tibial posterior slope of 3°.

To assess the influence of femoral flexion on the flexion gap, the tibial posterior slope was maintained at 3°, while the femoral flexion angle was incrementally adjusted from 3° to 8° in 1° intervals. Medial and lateral flexion gaps were recorded at each increment. Flexion gaps were measured at 90° of knee flexion, and extension gaps were measured in full extension (0°), as displayed by the robotic-assisted system interface. Similarly, to evaluate the effect of tibial posterior slope on knee gap dynamics, the femoral flexion angle was fixed at 3°, while the tibial posterior slope was adjusted from 3° to 7° in 1° increments. Medial and lateral flexion and extension gaps were documented for each setting.

**Fig. 1:**
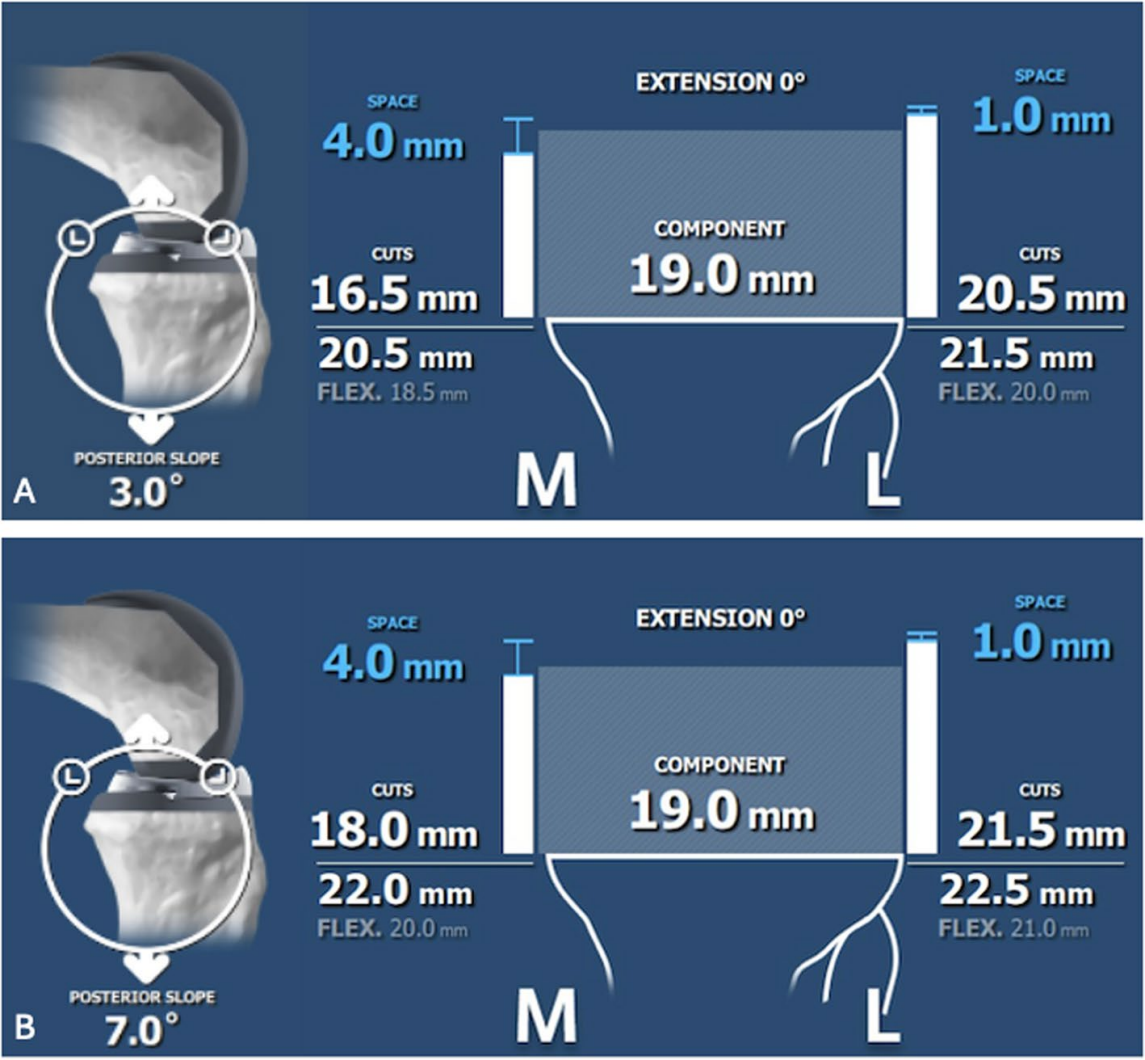
Effect of posterior tibial slope on gap measurements as displayed on the ROSA® Knee robotic system interface. **A** At a posterior tibial slope of 3°, **B** At a posterior tibial slope of 7

The femoral flexion angle and tibial posterior slope were adjusted using the ROSA® robotic system interface during the virtual surgical planning phase, and the corresponding gap measurements were recorded directly from the system’s planning screen. An example of this interface and the displayed measurements is shown in Fig. 1. This structured approach allowed for a comprehensive evaluation of the impact of sagittal alignment modifications on knee gap characteristics.

### Statistical analysis

All statistical analyses were conducted using R software (R Foundation for Statistical Computing, Vienna, Austria). To compare the medial and lateral flexion and extension gaps across different femoral flexion angles and tibial slope angles, Welch’s Two-Sample t-test was employed, accounting for unequal variances. Differences in gap measurements between flexion and extension positions at various tibial slope angles were further assessed using paired t-tests. The relationship between femoral flexion angle, tibial slope, and corresponding gap measurements was evaluated using Spearman’s rank correlation coefficient (ρ). Statistical significance was established at *P* < 0.05. Based on previous data by Okazaki et al. [14], which reported a mean flexion gap change of 1.9 mm with an SD of 0.6 mm, a paired-sample calculation with α = 0.05 and 80% power indicated that at least 10 subjects would be required to detect this difference. Our study included 40 patients, exceeding this minimum and providing sufficient power for the primary analysis.

## Results

The results demonstrated a clear trend of increasing medial and lateral gaps with higher femoral flexion and tibial posterior slope angles. Specifically, both medial and lateral flexion gaps gradually enlarge as femoral flexion increases from 3° to 8°. (Table 1) Similarly, both extension and flexion gaps show a corresponding increase as tibial posterior slope rises from 3° to 7°. (Table 2) Spearman correlation analysis, used to evaluate the relationship between femoral flexion angle, tibial slope, and gap measurements in TKA, indicated that both variables significantly influence gap dimensions. In particular, an increase in femoral flexion was associated with a proportional increase in both medial and lateral flexion gap﻿s (*ρ* > 0.99, *P* < 0.001), while a greater tibial slope correlated with increased medial and lateral gaps in both flexion and extension (*ρ* > 0.99, *P* < 0.001), suggesting a consistent effect on joint space across different knee positions.

**Table 1:** Flexion gap measurements at different femoral flexion angles

Femur flexion angle	Medial gap (Mean ± SD)	Lateral gap (Mean ± SD)
3°	14.53 ± 2.36	18.55 ± 2.63
4°	14.85 ± 2.32	18.74 ± 2.67
5	15.12 ± 2.31	19.38 ± 2.62
6°	15.47 ± 2.52	19.29 ± 2.65
7°	15.65 ± 2.53	19.61 ± 2.67
8°	15.88 ± 2.51	19.77 ± 2.70

**Table 2:** Knee gap measurements at different tibial posterior slopes

Tibia posterior slope	Medial gap (Mean ± SD)	Lateral gap (Mean ± SD)
**Extension gap**		
3°	14.15 ± 2.46	19.00 ± 2.95
4°	14.61 ± 2.47	19.24 ± 2.94
5°	14.89 ± 2.51	19.59 ± 2.99
6°	15.18 ± 2.49	19.86 ± 2.95
7°	15.58 ± 2.48	20.21 ± 3.03
**Flexion gap**		
3°	15.46 ± 2.51	19.27 ± 2.64
4°	15.87 ± 2.49	19.54 ± 2.66
5°	16.16 ± 2.54	19.89 ± 2.75
6°	16.43 ± 2.50	20.14 ± 2.78
7°	16.83 ± 2.50	20.50 ± 2.82

The analysis indicated that tibial slope influenced both flexion and extension gaps, with higher slopes generally resulting in slightly larger gaps. However, when adjusting the tibial slope, the changes in flexion and extension gaps occurred in equal proportion, with no significant difference between the two. Statistical testing revealed no significant proportional differences between flexion and extension gaps across any tibial slope angle (*P* > 0.05 for all comparisons). Table 3 presents a comparison of medial and lateral extension gap values alongside flexion gaps at tibial slope angles of 4°, 5°, 6°, and 7°, relative to a baseline tibial slope of 3°. Although there were minor variations in gap size as the tibial slope increased, the absence of significant differences suggests that tibial slope similarly impacts both gap types.

**Table 3:** Comparison of medial and lateral extension gaps with flexion gaps at different tibial slope angles, relative to a 3° baseline

Tibial slope	Comparison side	Mean difference from baseline (3° tibial posterior slope)		*P*-value	95% Confidence Interval
		**Extension gap**	**Flexion gap**		
4°	Medial	0.47	0.49	0.784	−0.08 to 0.106
4°	Lateral	0.31	0.35	0.5435	−0.087 to 0.164
5°	Medial	0.77	0.78	0.8506	−0.1223 to 0.148
5°	Lateral	0.65	0.69	0.5435	−0.087 to 0.164
6°	Medial	1.05	1.05	1	−0.135 to 0.135
6°	Lateral	0.92	0.95	0.7685	−0.147 to 0.199
7°	Medial	1.45	1.45	1	−0.158 to 0.158
7°	Lateral	1.27	1.3	0.7745	−0.152 to 0.203

## Discussion

Sagittal alignment plays a crucial role in TKA, influencing joint function, soft tissue balance, and the long-term success of implants. Surgeons can adjust the sagittal alignment of components to optimize knee mechanics [16]; however, the precise effects of these adjustments on joint space are still a topic of debate. [17, 18] While previous studies have explored various alignment strategies, there is limited quantitative data on how specific sagittal modifications affect medial and lateral gaps. To fill this gap, we analyzed knee gap dynamics across a range of femoral flexion angles and tibial slopes using a robotic-assisted system, which ensures high accuracy and reproducibility. Our results show that increasing femoral flexion and tibial slope leads to a progressive widening of both medial and lateral gaps. However, despite these changes, the differences in flexion and extension gaps across various tibial slopes were not statistically significant, suggesting that tibial adjustments have a consistent effect on joint space across knee positions.

Our study found that increasing the femoral flexion angle resulted in a progressive widening of both the medial and lateral flexion gaps. This finding contrasts with the results reported by Govardhan et al. In their navigated TKA approach, a mean femoral component flexion of approximately 5° was intentionally used to reduce the flexion gap by around 4 mm. They further noted that a 1.6° change in femoral flexion corresponded to a 1 mm reduction in the flexion gap [19]. The differences between our study and the technique described by Govardhan et al. likely stem from variations in surgical protocols, measurement methodologies, and technological platforms. In their navigated, tibia-first approach, soft-tissue releases are performed initially to equalize the medial and lateral extension gaps, and femoral component flexion is then applied as a secondary measure, specifically around the anterior cortical contact point, with immediate feedback on gap changes. In contrast, our robotic-assisted method systematically adjusts femoral flexion in controlled increments, focusing on high-precision gap measurements without relying as heavily on intraoperative soft-tissue balancing. These distinctions in technique and instrumentation may explain the observed differences in how femoral flexion impacts knee gap dynamics between the two studies.

Our study confirms the significant influence of tibial slope adjustments on both extension and flexion gaps, aligning with the findings of Nowakowski et al. and Okazaki et al. Consistent with Nowakowski et al., their study reported a gap increase of approximately 0.5–0.6 mm per degree of slope change in extension and 0.6–0.9 mm in flexion, [15] which closely corresponds to our findings. Furthermore, Okazaki et al. demonstrated that modifying the tibial slope by 5° led to a 1.8–1.9 mm change in the flexion gap for CR-TKA and 1.1–1.2 mm for PS-TKA [14], emphasizing the variation in tibial slope effects depending on implant design. Our results further support this trend, emphasizing the critical role of tibial slope adjustments in increasing the knee gap during total knee arthroplasty.

Both our study and the study by Nowakowski et al. demonstrate the influence of tibial slope on extension and flexion gaps in TKA [15]. However, there are notable differences in how tibial slope affects the proportional increase in these gaps. Nowakowski et al., using formalin-fixed knee joint specimens and CT scans, observed a more pronounced increase in the lateral compartment, particularly during flexion, with the lateral gap widening by approximately 30% more than the medial gap at each slope increment. They also found that the flexion gap increased more than the extension gap. In contrast, our study, which analyzed robotic-assisted TKA procedures with intraoperative data collection, standardized tibial resection angles, and fixed femoral flexion, found a more uniform increase in both flexion and extension gaps across tibial slope angles of 4°, 5°, 6°, and 7°, without significant differences between the two. These differences may be attributed to variations in methodology, such as the use of robotic-assisted technology in our study, compared to cadaveric specimens and CT imaging in Nowakowski’s study.

While this study assessed the effects of femoral flexion angle and tibial posterior slope independently, it is important to note that these parameters are frequently adjusted in combination during intraoperative gap balancing. Investigating the concurrent influence of both sagittal alignments on flexion and extension gap dynamics may yield a more comprehensive representation of clinical practice. Future studies are warranted to evaluate these combined effects and enhance the applicability of findings to real-world surgical scenarios.

This study has a few limitations. First, the relatively small sample size may have reduced the statistical power and generalizability of the findings. While the results are encouraging, a larger sample would provide more robust evidence and potentially uncover additional nuances in the relationship between sagittal alignment of the components and knee gaps. Second, the study relied exclusively on a computer navigation planning system, which may not fully replicate intraoperative conditions, potentially limiting the direct applicability of the findings to clinical practice. Third, although robotic-assisted TKA allowed for precise and reproducible measurements, variations in surgical technique and implant selection may have influenced the observed effects of sagittal alignment adjustments. Despite these limitations, the consistency of our findings with previous research supports their reliability. Future studies incorporating larger sample sizes and real-time intraoperative data would further strengthen the validity and clinical relevance of these conclusions.

## Conclusion

In conclusion, this study highlights the significant role of sagittal component alignment in gap balancing during total knee arthroplasty. Increased femoral flexion contributes to a larger flexion gap, while greater tibial slope results in proportional increases in both flexion and extension gaps. These findings provide valuable insights for surgeons to refine component positioning, optimizing joint stability and ligament tension. Future research incorporating intraoperative validation will be critical to further develop and personalize surgical strategies, ultimately enhancing patient outcomes in TKA.

## Data Availability

The datasets generated during this current study are available from the corresponding author upon reasonable request.
